# COVID-19 Beliefs, Self-Efficacy and Academic Performance in First-year University Students: Cohort Comparison and Mediation Analysis

**DOI:** 10.3389/fpsyg.2021.643408

**Published:** 2021-06-22

**Authors:** Kate Talsma, Kayleigh Robertson, Cleo Thomas, Kimberley Norris

**Affiliations:** School of Psychological Sciences, College of Health and Medicine, University of Tasmania, Hobart, TAS, Australia

**Keywords:** COVID-19, higher education, University, self-efficacy, confidence, academic performance, student, grades

## Abstract

Students’ learning contexts can influence their learning beliefs and academic performance outcomes; as such, students studying during the COVID-19 outbreak may be at risk of negative impacts on their academic self-efficacy and subject grades compared to other cohorts. They may also have specific beliefs about the impact of COVID-19-related changes on their capacity to perform, with potential consequences for self-efficacy and academic performance. Two weeks after the COVID-19-related transition to online-only learning, 89 first-year psychology students completed a measure of academic self-efficacy and indicated how they thought COVID-19-related changes would impact their capacity to perform in a psychology subject. At the end of the semester, subject grades were obtained from institutional records. Contrary to expectations, neither the self-efficacy beliefs nor the subject grades of the 2020 cohort were significantly different from those of a sample of 2019 first-year psychology students (*n* = 85). On average, 2020 students believed that COVID-19-related changes to their learning environment had a negative impact on their capacity to perform well. A mediation analysis indicated that students’ beliefs about the impact of COVID-19 on their capacity did not directly, or indirectly (*via* self-efficacy), predict grades. The only significant association in the model was between self-efficacy and grades. Although students reported believing that COVID-19-related changes would negatively impact their capacity to perform, there is little evidence that these beliefs influenced their academic self-efficacy or academic performance or that studying during the COVID-19 outbreak disadvantaged students in comparison with the previous years. A follow-up analysis indicated that self-efficacy was a stronger predictor of grades in the 2020 cohort than in the 2019 cohort. While there may be several unmeasured reasons for cohort differences, one potential interpretation is that, in the context of uncertainty associated with COVID-19, self-efficacy beliefs assumed relatively greater importance in terms of mobilising the resources required to perform well.

## Introduction

COVID-19, the first coronavirus to be declared a global pandemic (World Health Organization, 2020), has caused unprecedented disruption in many life domains. In order to reduce the spread of coronavirus, many universities around the world made an abrupt transition from face-to-face to online and remote learning (Ali, 2020; Aristovnik et al., 2020; Centers for Disease Control and Prevention, 2020; Coyne et al., 2020). Many institutions currently continue to either teach fully online or use hybrid models (Walke et al., 2020). Students in higher education are considered a particular risk group for COVID-19-related impacts (Xiong et al., 2020). In this context, university students who have been studying during the COVID-19 outbreak are potentially vulnerable to negative effects on academic outcomes, such as grades, as well as on important academic beliefs, such as self-efficacy.

COVID-19-related changes have disrupted students’ lives in many ways. Research reviewed by Aristovnik et al. (2020) indicates that students have experienced changes to their habits and daily routines, reduced social contact and support, and financial impacts. Campus closures have decreased access to libraries and other face-to-face supports (Patricia Aguilera-Hermida, 2020), as well as to internet facilities, printers, and other essential equipment and services (Aristovnik et al., 2020). Many students’ new at-home study environments are not conducive to focused work, often being shared spaces characterised by noise and distractions (Patricia Aguilera-Hermida, 2020). In fact, more than half of students in a global study reported that they did not have a quiet place to study (Aristovnik et al., 2020).

Students who had to abruptly and involuntarily transition to online learning may not have been well equipped to function successfully in their new learning environment. The need to adapt to an unanticipated – and perhaps undesired – way of learning may impact performance outcomes because of a lack of confidence in, certainty about or acceptance of, online learning (Mäkitalo et al., 2005; Tarhini et al., 2017; Sollitto et al., 2018; Bower, 2019). Underdeveloped self-regulation skills may also be a concern, given how important self-regulation skills are for online learning generally (Broadbent and Poon, 2015; Aristovnik et al., 2020) and that online learning potentially requires the application of different types of self-regulation skills compared to face-to-face study (Broadbent, 2017). Students may also have lacked the time management and IT skills to engage effectively with learning materials delivered in the online format (Aristovnik et al., 2020; Patricia Aguilera-Hermida, 2020). Students have also reported that they anticipated less success in communicating with their teachers and classmates as a result of remote learning (Patricia Aguilera-Hermida, 2020), which is of concern in the COVID-19 context given that students rely on peer communication to manage academic uncertainty (Sollitto et al., 2018) and the role that social support and information sharing have in facilitating student resilience and adaptation (Wilks, 2008).

Alongside these practical impacts on students’ learning experiences, public health emergencies also carry widespread mental health implications. Research indicates that the outbreak of COVID-19 has been accompanied by negative psychological effects, including increased feelings of stress, and increased symptoms of anxiety and depression (Brooks et al., 2020; Salari et al., 2020; Taylor et al., 2020). In many cases, these effects are believed to reach thresholds for clinical significance in the general population (Xiong et al., 2020). High levels of uncertainty are associated with academic stress (Akgun and Ciarrochi, 2003) and a higher prevalence of mental disorders (Wu et al., 2020), both of which are negatively predictive of academic performance (Bewick et al., 2010; Bedewy and Gabriel, 2015). Students around the world surveyed during COVID-19 lockdowns have reported increased stress, anxiety and worries, as well as boredom, frustration and a lack of motivation (Aristovnik et al., 2020; Cao et al., 2020; Patricia Aguilera-Hermida, 2020). For example, Liu et al. (2020) reported that Chinese students’ anxiety and depression levels during the COVID-19 outbreak were higher than national norms. Research by Lechner et al. (2020) in the United States indicated that students’ alcohol consumption increased following university closures. A majority of French students surveyed during a period of COVID-19-related confinement reported increased anxiety and moderate-to-severe stress (Husky et al., 2020). COVID-19, like other public health epidemics, is seen as a chronic stressor, potentially resulting in significant changes to thoughts, feelings and behaviours (Liu et al., 2020) across multiple domains of functioning, including the academic context.

While some degree of arousal is beneficial for learning, meta-analyses have shown that stress, depression and anxiety have a negative relationship with memory and academic performance overall (Seipp, 1991; Kizilbash et al., 2002; Richardson et al., 2012). Alongside the practical impacts of COVID-19 on learning experiences described above, it may also be anticipated that students’ academic performance will suffer as a result of the psychological impacts of the COVID-19 outbreak. Commentary regarding the potential impact of COVID-19 on learning outcomes paints a bleak picture consistent with this prediction (e.g. Aucejo et al., 2020; Dorn et al., 2020). However, empirical findings are mixed in this regard. For example, one study with United States students showed that, although students expected to perform more poorly and reported decrements in terms of knowledge, concentration and engagement, actual grades were unchanged (Patricia Aguilera-Hermida, 2020). Indeed, it has even been reported that academic performance in higher education is positively influenced by COVID-19, with Spanish students impacted by COVID-19 performing better than students in a previous cohort (Gonzalez et al., 2020). Further research is needed to explore how COVID-19 has impacted academic performance outcomes in additional samples.

The COVID-19 pandemic also has potential implications for students’ academic beliefs. Self-efficacy, a key construct in social cognitive theory, is an individual’s “can do” belief about a future performance outcome (Bandura, 1997). In academic settings, self-efficacy is widely believed to be one of the most important non-intellective predictors of achievement. Meta-analyses of the relationship between self-efficacy and academic performance consistently demonstrate a positive association between the two (e.g. Multon et al., 1991; Honicke and Broadbent, 2016). For example, Richardson et al. (2012) identified self-efficacy as the strongest correlate (*ρ* = 0.59) of grade point average (GPA) from 42 non-intellective antecedents of performance from 13 years of research. A systematic review of meta-analyses showed that self-efficacy was the strongest student-related predictor of achievement in higher education (*d* = 1.81; Schneider and Preckel, 2017). Other meta-analyses have shown that self-efficacy remains a positive, albeit modest, predictor of academic performance when previous academic performance is taken into account (Valentine et al., 2004; Talsma et al., 2018).

A key issue in terms of the impact of COVID-19 on self-efficacy beliefs is that emotional states are identified as a source of these types of judgements (Bandura, 1997). Individuals are believed to use their own feelings of arousal, uncertainty, anxiety, stress and fatigue as cues in judging their own efficacy (Usher and Pajares, 2006). For example, low levels of arousal (feeling calm) may be interpreted as compatible with a sense of personal competence. In contrast, feelings of distress or other strong negative emotions regarding academic tasks can undermine beliefs about capability and decrease performance expectations. As such, a student experiencing study-related distress associated with COVID-19-related changes to their learning context may interpret this distress as an indication of vulnerability to perform poorly (Bandura, 1995). It is well established that academic self-efficacy and anxiety are negatively related (Pintrich and De Groot, 1990; Rouxel, 1999), with both meta-analyses (Preiss et al., 2006) and recent research in the COVID-19 context supporting this pattern of association (Alemany-Arrebola et al., 2020). With a growing number of studies showing heightened anxiety (as well as depression, frustration and boredom) during the COVID-19 outbreak (Aristovnik et al., 2020; Cao et al., 2020; Patricia Aguilera-Hermida, 2020), there is the potential for self-efficacy beliefs to be negatively impacted. While some research suggests that self-efficacy beliefs are resistant to external influences (e.g. Bong, 2002; Foster et al., 2016), there is also evidence that they can change in response to interventions (e.g. Bergey et al., 2019) and over time (e.g. Talsma et al., 2020). As such, we anticipated that the unprecedented practical and psychological impacts of COVID-19 would be reflected in lower academic self-efficacy beliefs for 2020 university students than comparable cohorts.

There is some emerging evidence to suggest this may well be the case. When facing the challenges outlined above, students have reported expecting to perform more poorly in their academic work because of the impact of COVID-19 (Aucejo et al., 2020; Patricia Aguilera-Hermida, 2020). For example, more than 50% of students at a United States university expected their GPA to be negatively impacted by COVID-19 (Aucejo et al., 2020), while only 7% of students believed their grades would be positively impacted. Similarly, students from another US university surveyed during the COVID-19 pandemic reported that, compared to before the outbreak, they expected to perform more poorly in their classwork and have more difficulty submitting assignments on time (Patricia Aguilera-Hermida, 2020). While students have reported that they expect to face academic challenges as a result of COVID-19, there is no study to our knowledge which has measured self-efficacy beliefs during COVID-19 using a standardised instrument and compared scores to an analogous previous cohort.

The current study contributes to an emerging field of research exploring the impact of COVID-19 on the university sector. Limited research has explored how the distressing context of COVID-19 may impact university students’ self-efficacy beliefs. Likewise, the practical and psychological implications of COVID-19 may impact students’ academic performance outcomes. While a very small number of studies have considered relationships between self-efficacy and other variables during COVID-19 (e.g. Alemany-Arrebola et al., 2020), none, to our knowledge, compared the self-efficacy beliefs of students impacted by COVID-19 to those of students not impacted by COVID-19. At the same time, commentary and research around the impact of COVID-19 on academic outcomes have yielded mixed results, and additional research is needed. Thus, the first aim of the current study was to compare the self-efficacy beliefs and academic performance outcomes of 2019–2020 cohorts of students studying the same course. It was hypothesised that the self-efficacy beliefs and subject grades of 2020 students would be significantly lower than those of 2019 students.

Further to the above aims, it is also important to explore how 2020 students’ beliefs about the impact of COVID-19 on their performance capacity relate to other key academic variables. A small number of previous studies have examined student beliefs regarding the impact COVID-19 will have on their performance (Aucejo et al., 2020; Patricia Aguilera-Hermida, 2020), but there are no studies, to our knowledge, which assess how these specific beliefs predict broader self-efficacy judgements or academic performance outcomes, such as grades. Therefore, the second main aim of the current study was to conduct such an investigation. It was hypothesised that COVID-19 beliefs would predict academic performance outcomes, both directly, and indirectly *via* their influence on self-efficacy beliefs.

## Materials and Methods

### Participants

Participants were 89 first-year undergraduate students (69 female) studying introductory psychology in semester 1, 2020 at a regional Australian university. Participants’ ages ranged from 18 to 51, with an average age of 23.5 years. The sample was predominantly Australian (76 students).

Data from the participants in the 2019 comparison cohort (85 students, 60 female) had been collected for a previous research project. These students were also studying introductory psychology in semester 1. Participants’ ages ranged from 18 to 64 (*M* = 24.6). This sample was also mostly Australian (75 students).

### Procedure and Measures

Participants were invited by email to complete an online questionnaire containing the below measures, which took approximately 15 min to complete. The online questionnaire was open for a period of 10 days in early April 2020, beginning approximately 2 weeks after COVID-19-related teaching and learning changes (e.g. transition to online classes) began. The Human Research Ethics Committee of the authors’ institution reviewed and approved the project. Participants received course credit for participating.

Self-Efficacy was measured using a 7-item scale adapted from the Motivated Strategies for Learning Questionnaire (MSLQ) self-efficacy subscale (Pintrich and De Groot, 1990). Participants indicated their perceived capability of overall performance in their specified subject on a 7-point Likert scale ranging from 1 = not at all true of me to 7 = very true of me (e.g. “I am confident in my ability to receive an excellent grade in this class”). The MSLQ has demonstrated good predictive validity of future academic performance (Pintrich et al., 1993) and original psychometric testing showed a Cronbach’s *α* of 0.93 (Pintrich and De Groot, 1990).

A single item asked participants to indicate what impact they believed the COVID-19-related changes to their university context would have on their capability to perform in their studies. Participants answered on a 5-point semantic-differential scale ranging from 1 = strongly negative impact to 5 = strongly positive impact.

Academic performance scores reflected students’ subject grades (0–100) in an introductory psychology subject. Subject grades were based on scores on tests/exams as well as on assignments assessed using standardised assessment rubrics. Participants provided their consent for questionnaire responses to be matched with grades obtained from institutional records.

Participants also indicated their age, sex and nationality.

### Power

While our primary goal in the circumstances was to recruit as many participants as possible within the limited window available, we considered the following in terms of our sample. For mean differences in self-efficacy and academic performance between the 2019 and 2020 cohorts, a calculation using G*Power 3.1 (Faul et al., 2007) indicated that a sample size of 60 participants per group would be required to detect medium effects. For the mediation analysis, the recommendation to recruit a sample of at least 50 participants plus 8 per variable suggested a sample size of at least 74 participants (Tabachnick et al., 2007).

## Results

All analyses were conducted in jamovi (The jamovi project, 2020). Preliminary independent-samples *t*-tests indicated that the self-efficacy, COVID-19 beliefs and academic performance outcomes of Australian students did not differ significantly from those of students with other nationalities, and there were no differences between males and females. For the self-efficacy scale, both Cronbach’s *α* and McDonald’s *ω* (argued to be a better alternative; Peters, 2014; Deng and Chan, 2017) for the current sample were 0.93.

Table 1 shows the means, standard deviations and correlation matrix for the key study variables for 2020 students. There was no final grade data available for four students; these students were excluded pairwise from analyses involving grades.

**Table 1:** Correlation matrix, means (SDs) on the diagonal.

	COVID-19 beliefs	Self-efficacy	Academic performance
COVID-19 beliefs	2.09 (0.98)		
Self-efficacy	0.122	5.08 (1.01)	
Academic performance	0.088	0.294^**^	71.9 (17.30)

** *p* < 0.01.

### COVID-19 Beliefs

A one-sample *t*-test comparing the mean COVID-19 beliefs score to a test value of three (reflecting the scale midpoint value of neutral impact) indicated that students believed, on average, that COVID-19-related changes to their learning context would have a negative impact on their capacity to perform, *t*(88) = −8.72, *p* < 0.001, with a large effect, *d* = 0.925. Of the whole sample, 52 students (81.6%) believed that the COVID-19-related changes would negatively impact their capacity to perform [22 students (24.7%) strongly so]. Five students (5.7%) indicated that they believed COVID-19 would have no effect on their capability, while 11 students (12.6%) believed that the changes would have a positive impact on their ability to perform [three students (3.4%) strongly so].

### 2019 and 2020 Cohort Comparison

The self-efficacy beliefs of students in the 2020 cohort did not differ significantly from those in the 2019 cohort (*M* = 4.76, *SD* = 1.22), *t*(172) = 1.859, *p* = 0.065. We note that Cohen’s d for this test indicated a small effect size (*d* = 0.282), with mean self-efficacy scores of 2020 students exceeding those of 2019 students, contrary to expectations. Grades for 2020 students also did not differ significantly from those of 2019 students (*M* = 73.31, *SD* = 17.34), *t*(167) = 0.650, *p* = 0.516, *d* = 0.10. Mann-Whitney *U*-tests were also conducted to address potential concerns regarding violation of assumptions of the Student’s *t*-tests; non-parametric test results were substantively consistent with the above (Self-efficacy: *U* = 3,155, *p* = 0.059; Grades: *U* = 3,312, *p* = 0.417).

### Mediation Analysis

**Figure 1:**
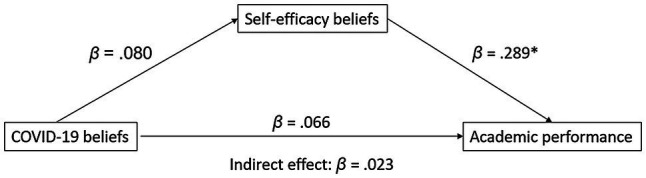
Prediction of academic performance by COVID-19 beliefs *via* self-efficacy. ^*^p < 0.05.

As shown in Figure 1, students’ beliefs about the impact of COVID-19 on their performance capacity did not predict overall academic self-efficacy beliefs (*z* = 0.777, *p* = 0.437), nor did they directly (*z* = 0.740, *p* = 0.459) or indirectly (*z* = 0.734, *p* = 0.463) predict academic performance outcomes. The only significant relationship in the model was the prediction of academic performance by self-efficacy (*z* = 2.376, *p* = 0.018).

### Comparison of Correlations

As a follow-up, we conducted an unplanned comparison of the correlations between self-efficacy and academic performance across cohort years. For the 2019 sample, there was no significant correlation between self-efficacy and academic performance, *r*(85) = 0.172, *p* = 0.116. For the 2020 sample, there was a moderate positive correlation, *r*(86) = 0.413, *p* < 0.001. We investigated the differences between correlations using the cocor package (Diedenhofen and Musch, 2015) in R (R Core Team, 2013). There was no statistically significant difference between the two correlations, *z* = −1.692, *p* = 0.091 (two-tailed). Also, the 95% of the confidence interval for the difference between the two correlations (−0.508, 0.038) included zero (Zou, 2007). Thus, we could not reject the null hypothesis of no significant difference between the correlations. However, interpreting the difference between the two *r*-to-*Z* transformed correlations yielded a Cohen’s *q* of 0.265, suggesting a medium effect for this comparison, and we note that a G*Power analysis (Faul et al., 2007) suggested a much larger sample size (*n* = 178 in each group) would have been necessary for an effect of this size to be deemed statistically significant.

## Discussion

This study compared the self-efficacy beliefs and subject grades of the 2020 cohort of introductory psychology students which was impacted by COVID-19 with those of a comparable 2019 cohort. Contrary to expectations, there were no significant differences between the two cohorts on these two variables. We further anticipated that 2020 students’ beliefs about the impact of COVID-19 on their capacity to perform would inform self-efficacy beliefs and also predict academic performance directly and indirectly (*via* self-efficacy). While students believed, on average, that COVID-19-related changes to their learning environment would have a negative impact on their capacity to perform, these beliefs did not predict either self-efficacy more broadly or subject grades.

### Interpretation: Academic Performance

It had been anticipated that the practical and psychological upheaval associated with COVID-19 would be associated with poorer academic outcomes in the 2020 cohort. It was also expected that beliefs about the impact of COVID-19 would predict academic outcomes. It is not possible to determine exactly why these expectations were not supported, but the following considerations may be relevant. On the one hand, it is possible that the anticipated negative impact of COVID-19 on academic performance was simply not borne out in our sample. Our findings are consistent with the small amount of previous research which suggests that students have negative expectations related to COVID-19 specifically, but that these are not necessarily matched by objective academic performance outcomes (Gonzalez et al., 2020; Patricia Aguilera-Hermida, 2020). One interpretation of this is that COVID-19-related changes were not sufficiently impactful to influence performance outcomes. While predictors of academic success have been of interest to psychology and education researchers for more than a century, much research suggests that academic performance remains consistent over time (Talsma et al., 2018, 2019a) and that stable factors, such as genes, demographics, socio-economic status, parental educational background, intelligence and prior achievement, are the primary predictors of academic outcomes (Stegers-Jager et al., 2015; Rimfeld et al., 2018; von Stumm et al., 2020). While research regarding a wide range of malleable psychosocial predictors of academic performance has also proliferated, scholars have recently cautioned against overstating the potential impact that environmental differences and non-cognitive factors can have on individual capacities and real-world outcomes (Moreau et al., 2019). In the context of strong predictions of academic outcomes by the stable factors outlined above, it may simply be that the COVID-19-related changes to students’ learning environment did not reach an impact threshold whereby academic performance outcomes were affected. This may also explain why COVID-19 beliefs, in spite of their salience to students, were independent of objective performance results in our sample. Our findings are also consistent with the previous research showing that academic outcomes are not differentiated by course delivery mode (e.g. Cavanaugh and Jacquemin, 2015).

On the other hand, in terms of the finding of no grade differences between cohorts, there is a range of plausible alternative explanations. For example, it is not possible to rule out the potential influence of steps taken at an institutional level to buffer the anticipated negative impact of COVID-19 on students’ experiences. In the case of the institution where this research was conducted, for example, a COVID-19-specific policy was in place to provide more flexibility around applications for extensions to assignment deadlines. In the absence of such policies, academic performance outcomes may have suffered. It is also possible that unmeasured changes, such as the amount of effort expended on studying, could have influenced these results. It is plausible that a desire to reduce anxiety led to increased effort devoted to studying, or that a reduction in social activities or un-/under-employment associated with COVID-19-related changes meant that more time was available for students to study. There is some evidence that COVID-19 impacted the number of time students spent studying, with some studying much more than usual, and others studying much less (Aucejo et al., 2020). It is thus possible that, while average grades could not be differentiated across cohorts, individuals responded to the situation differently, with impacts on who exactly received what grade.

### Interpretation: Self-Efficacy

As with academic performance, there was no evidence of cohort differences in self-efficacy beliefs. This was contrary to expectations and inconsistent with reports of students’ diminished performance expectations during COVID-19 lockdowns (e.g. Aucejo et al., 2020; Patricia Aguilera-Hermida, 2020). Potential explanations for this lack of effect mirror those regarding academic performance in many respects. For example, while self-efficacy is theoretically domain- and context-specific and thus considered to be subject to variations within individuals and over time (Bandura, 1997; Bong and Skaalvik, 2003), there is some evidence that established self-efficacy beliefs are resistant to change and may not be as responsive to contextual determinants as theory suggests, as noted above (Bong, 2002). Several studies suggest that when beliefs about academic performance capacity are measured on multiple occasions over time, even when there are many opportunities for feedback and monitoring in-between measurements, correlations between repeated measurements are consistently strong (Bong, 2001, 2002; Hacker et al., 2000, 2008; Foster et al., 2016). In support of this, a meta-analytic cross-lagged panel analysis of the relationships between self-efficacy and academic performance over time showed that the previous self-efficacy was a very strong predictor of subsequent self-efficacy (Talsma et al., 2018). The present findings suggest that self-efficacy beliefs may not be strongly impacted even in the case of extreme changes to external circumstances; however, we note that a within-subjects design would be needed to confirm this. It has also been noted that university students are often overconfident with regard to their self-efficacy beliefs (Talsma et al., 2019b, 2020). As such, it is possible that the anticipated impact of COVID-19 was insufficient to disrupt students’ tendencies to express strong confidence in their capacities. Another potential explanation for this unexpected finding is that part of the rationale for hypothesising lower self-efficacy in the 2020 cohort rested on the fact that emotional states and moods are a theorised source of self-efficacy beliefs. In the context of established negative psychological impacts of COVID-19, this expectation was defensible. However, in empirical studies, emotional states and physiological arousal tend to show a negligible or trivial influence on self-efficacy beliefs, while mastery experiences or performance accomplishments – which are, again, more stable – are believed to be the primary source of such judgements (Usher and Pajares, 2008; Phan, 2012). The lack of differences observed across cohorts, and the lack of influence of beliefs about COVID-19 on self-efficacy suggest that established self-efficacy beliefs tend to be stable and are not strongly influenced by changes in context.

Having said that, we noted above that institution-level response to the COVID-19 crisis may have impacted students’ academic performance outcomes, and there is also the possibility that students’ self-efficacy beliefs were buffered by such policies, which were implemented and communicated to students shortly prior to data collection for this study. However, if this was the case, such buffering would likely have also influenced responses to the question about the impact of COVID-19-related changes on the capacity to perform. We note that, in the structure of the questionnaire, students were asked about the impact of COVID-19 after they were asked about self-efficacy. The reason for this is that it was believed that, by bringing the potential threat of the pandemic to students’ minds, the COVID-19 question could introduce expectancy effects that might influence subsequent responses. It may be that COVID-19 beliefs and self-efficacy beliefs were unrelated because students’ self-efficacy judgements were made independently of thoughts about COVID-19 and based on traditional patterns of responding which emphasise the stability of self-efficacy and overconfidence as outlined above. Meanwhile, it is possible that making COVID-19 salient to students prompted defensive pessimism, proactive external attributions or anticipatory cushioning to protect self-worth against possible negative outcomes (Norem and Cantor, 1986a,b; Campbell and Sedikides, 1999; Sedikides et al., 2004; Weiner, 2010). As with academic performance outcomes, we note that it is possible that we saw no difference in mean self-efficacy beliefs across cohorts because some students’ beliefs were negatively impacted by the COVID-19 context, while others were positively impacted. For example, the self-efficacy beliefs of some students may have decreased in line with our hypothesis, but institutional and teacher communications with students designed to provide reassurance may have buffered the beliefs of other students, considering that verbal persuasion is also a source of self-efficacy beliefs (Bandura, 1997; Usher and Pajares, 2008).

### Interpretation: Relationship Between Self-Efficacy and Academic Performance

The finding that self-efficacy was positively predictive of grades in the path analysis is consistent with previous research as outlined above. It was also found that the correlation between self-efficacy and academic performance was larger in the 2020 cohort than in the 2019 cohort. While this difference was not statistically significant, we note that the analysis was underpowered, and the effect size was medium in magnitude. Thus, while further evidence is needed, it appears plausible that a meaningful effect may exist. One potential interpretation of this is that, when faced with the pandemic situation, self-efficacy beliefs exerted an influence on how students appraised the potential impact of COVID-19 (e.g. fostering an appraisal of challenge rather than threat) and on choices they made about how to engage with their learning (e.g. prompting adaptive learning behaviours like setting high goals; Bandura, 1997). In the “business-as-usual” case of the 2019 cohort, self-efficacy beliefs may have exerted a lesser influence.

We note that the sizes of the effects of the self-efficacy/performance relationship described above do not take into account cognitive ability or previous academic performance. The size of the effects would likely be attenuated if this had been the case, because in the reciprocal relationship between self-efficacy and academic performance, the unique effect of self-efficacy on performance is small (Valentine et al., 2004; Talsma et al., 2018, 2019a).

### Limitations

There are a number of limitations which impact the interpretation of findings in the present study. For the most part, these relate to unavoidable issues associated with the COVID-19 situation and with comparing different cohorts of students.

First of all, in order to begin collecting data in as timely a fashion as possible, we prepared and sought urgent ethical approval for a single-item measure of students’ beliefs about the impact of COVID-19 on their capacity to perform academically. Single-item measures are subject to criticism (e.g. Diamantopoulos et al., 2012); however, we note that recent studies support their validity and utility in research (e.g. Hoeppner et al., 2011).

The need to collect data within a specific time period during the COVID-19 shutdown limited the sample size we were able to recruit. Our inability to reject null hypotheses in both the cohort comparison and mediation analysis may suggest that insufficient power is a concern. Our power analyses were based on medium effects; however, several small or trivial effects were noted and these would not have reached statistical significance without considerably larger sample sizes. However, we note that the small self-efficacy effect that we reported is in the opposite direction to that hypothesised, which provides some indication that the COVID-19 context in 2020 was not accompanied by a *negative* impact on self-efficacy beliefs. In terms of the mediation model, while the trivial effects of COVID-19 beliefs on self-efficacy and academic performance may have reached statistical significance with a much larger sample, it seems unlikely that effects of the magnitude identified (i.e. *β* = 0.080 and smaller) would be considered meaningful.

A limitation of cohort studies, in general, is the potential for influences of unmeasured confounding variables. This concern is potentially compounded in the present case, as the pandemic situation was evolving rapidly at the time data was collected. It is not possible to explicitly determine the effect of COVID-19 on outcomes using the between-group cohort comparison design necessitated by the real-world setting of this research. While this approach has some benefits in comparison with studies which have asked students to retrospectively self-report pre-COVID-19 feelings, beliefs and behaviours, our findings should be interpreted with caution. For example, while assessments in both cohorts were either marked by computer or based on standardised rubrics and subject to moderation, it is unknown to what degree assessors consciously or unconsciously adjusted their marking practices to take into account anticipated negative impacts of COVID-19 on performance. As well as the possible impact of unknown differences between cohorts, we also note that there were known differences between cohorts which cannot be quantified in our analysis. For example, while subject content and teaching staff remained the same across the two cohorts, there were changes in assessment between 2019 and 2020 (e.g. written assignment topic change, minor assessment weighting changes and move to online exams because of COVID-19 distancing requirements). COVID-19-related institutional policy changes have also been referred to above.

Several issues may also have impacted the data and we had available for analysis. For example, it is possible that students for whom final grades were unavailable could have received lower grades, reducing the average performance score. We focused on a single subject of study, but it is possible that participants may have received higher or lower grades in other subjects, or their courses overall. There is also a potential risk that students with lower self-efficacy beliefs and poorer grades did not participate in the study. For example, students experiencing the greatest negative impacts of COVID-19 may not have volunteered to participate in the study or may have already withdrawn from the subject when questionnaire invitations were sent. We note, however, that students in all cohorts experience stresses and challenges; the 2019 sample may have been influenced by similar issues as well. One other point to consider is that the use of identical measures for self-efficacy in both cohorts may mean that scores are subject to common method bias (see e.g. Spector, 2006).

Some comments are merited regarding the characteristics of the present sample, as they may affect the interpretation and generalisability of these findings. Our sample was potentially, especially vulnerable to negative impacts of COVID-19 because of its makeup. For example, participants were mostly female, mostly young adults and, naturally, all students. All of these characteristics have been identified as risk factors for negative outcomes relating to COVID-19 (Xiong et al., 2020). In addition, the university where the research was conducted is located in the southern hemisphere, where university students have been identified as potentially particularly susceptible to negative outcomes, given that the lockdown began early in the new academic year for these students (Aristovnik et al., 2020). Several scholars have also noted the potential difference in COVID-19 impacts depending on socio-economic status, which may result in the exacerbation of digital and economic inequalities in student populations (Aristovnik et al., 2020; Dorn et al., 2020). While the institution where this research was conducted is located in a western and industrialised nation, the specific location is a regional area identified as having the highest proportion of people living in the most disadvantaged areas of the country, and vice versa (Australian Bureau of Statistics, 2016). Thus, while we note several potential limitations to the present research above, in the context of anticipated vulnerabilities of the sample, the lack of significant findings may be considered noteworthy. Conversely, we note that the particularities of the present sample may limit the generalisability of findings to similar student samples.

### Conclusion

Overall, while 2020 students believed that COVID-19-related changes to their learning context would negatively impact their capacity to perform academically, neither their self-efficacy beliefs nor their academic performance outcomes differed from a comparable 2019 cohort. Furthermore, 2020 students’ beliefs about COVID-19 did not directly predict performance outcomes or self-efficacy beliefs, nor did they indirectly predict performance outcomes *via* self-efficacy. This provides some comfort that perhaps not all of the bleak predictions associated with COVID-19 will be borne out; both subject grades and self-efficacy beliefs may have been buffered by policies with this end in mind, or they may more generally be resistant to the impact of variations in the environmental context.

## Data Availability Statement

The raw data supporting the conclusions of this article will be made available by the authors, without undue reservation.

## Ethics Statement

The studies involving human participants were reviewed and approved by the Human Research Ethics Committee of the University of Tasmania. The patients/participants provided their written informed consent to participate in this study.

## Author Contributions

KT conceived of the project with input from KN. KR and CT undertook data collection under the supervision of KT. KT conducted statistical analyses and wrote the manuscript with input from KR and KN. All authors contributed to the article and approved the submitted version.

### Conflict of Interest

The authors declare that the research was conducted in the absence of any commercial or financial relationships that could be construed as a potential conflict of interest.
